# Criterion-Based Audit of Hand Hygiene Performance During Caesarean Section at a Referral Hospital in Northern Tanzania: An Uncontrolled Interventional Study

**DOI:** 10.24248/EAHRJ-D-19-00014

**Published:** 2019-11-29

**Authors:** Enna Sengoka, Lærke Rasmussen, Marycelina Msuya, Godfrey Kisigo, Bjarke Lund Sørensen, Jaffu Chilongola, Eusebious Maro

**Affiliations:** a Kilimanjaro Christian Medical University College, Moshi, Tanzania; b Kilimanjaro Christian Medical Centre, Moshi, Tanzania; c Bjelke Allé 22,01,20, 2200, Copenhagen, Denmark; d Kilimanjaro Clinical Research Institute, Moshi, Tanzania; e Department of Clinical Medicine, University of Copenhagen, Copenhagen, Denmark

## Abstract

**Background:**

Health-care-associated infection (HCAI) is a big challenge in both low- and high-income countries. Around childbirth, infection is among the main causes of maternal and perinatal morbidity and mortality. Appropriate hand hygiene practice is a simple and cost-effective way of reducing HCAIs. This study aimed to assess the baseline performance and knowledge of proper hand hygiene during caesarean sections and the impact of interventions guided by a criterion-based audit at a tertiary health facility in Tanzania.

**Methods:**

A noncontrolled, before-and-after intervention study, guided by a criterion-based audit, was carried out. A criterion based checklist was used for direct observations of hand hygiene performance during cesarean section. A self-administered questionnaire was used to assess knowledge on infection prevention. Performance was compared before and after a half-way intervention.

**Results:**

At baseline, low-quality hand hygiene performance was observed. Significant improvements of hand hygiene performance were observed for a number of criteria. Long nails: performance reduction from 15 (25%) to 3 (5%) (*P*=.04), polished nails: from 11 (18%) to 1 (2%) (*P*=.04), a score increase in hand wash with water from 43.8 (73%) to 60 (100%) (*P*=.001). Postoperatively, correct glove removal increased from 20 (33%) to 37.8 (66%) (*P*=.01). Alcohol-based hand rub use increased from 2 (3%) to 21 (35%) (*P*=.001). The number of health-care workers who did not wash hands after procedure with either water or alcohol-based hand rub reduced from 35 (58%) to 10 (17%) (*P*=.001). After the intervention, poor knowledge among health-care workers reduced from 7 (39%) to 3 (17%), while moderate knowledge increased from 8 (44%) to 12 (67%).

**Conclusion:**

Feedback, discussion of findings, training, visual reminders, and distribution of alcohol-based hand rub, as part of a criterion-based audit is a powerful way of improving hand hygiene performance and knowledge in surgical wards.

## INTRODUCTION

Health-care-associated infections (HCAIs) are infections not present at the time of admission but acquired in the process of patient care.^1^ The impact of HCAIs is significant, since not only by their effects on patients by increasing morbidity and mortality, but also prolonged hospital stay, enhance development and spread of resistance of microorganisms to antibiotics, and higher economic losses.^2–4^ The global burden remains unknown because of the difficulty of collecting reliable data. However, estimates shows that hundreds of million people worldwide are afflicted by infections acquired in hospitals.^5^

The prevalence of HCAIs is considered higher in low- and middle-income than in high-income countries, particularly in patients admitted to intensive care and neonates units.^1,6^ Prevalence of HCAIs in developing countries ranges from17% to 19%^5^ while in Africa it is reported to range from 3% to 15%.^7,8^ The prevalence in Sub Saharan Africa ranges from 2% to 29%,^9^ whereas in Tanzania, reports show a prevalence of 15%.^10^ The most frequent maternal HCAIs are urinary tract infection, endometritis, chorio-amnionitis and infection due to operative and vaginal birth. Women who have caesarean sections are more likely to become infected than women who deliver vaginally.^11,12^

Hand hygiene has long been considered the central tenet of infection prevention aimed at limiting the spread of HCAIs and Multi Drug Resistant Organisms (MDROs) as well as susceptible pathogens. However, despite knowledge of the importance of hand hygiene in prevention of HCAIs, compliance is notoriously poor. Known risk factors for HCAIs in women are poor knowledge and application of basic infection control (IC) measures, hereunder good hand hygiene, furthermore prolonged and inappropriate use of invasive devices and antibiotics, understaffing and insufficient equipment.^1^ Delivery complications such as prolonged labor, prolonged membrane rupture, multiple vaginal examinations and manual removal of the placenta are additional risk factors.^1,5,12^ Microorganisms responsible for HCAIs can be viruses, fungi, parasites and bacteria such as *Staphylococci, Klebsiela, Candida albicans* and *E. coli* which can be present at the patient's skin, transmitted from another patient or from the surrounding environment or health-care worker.^9^

**FIGURE 1.**
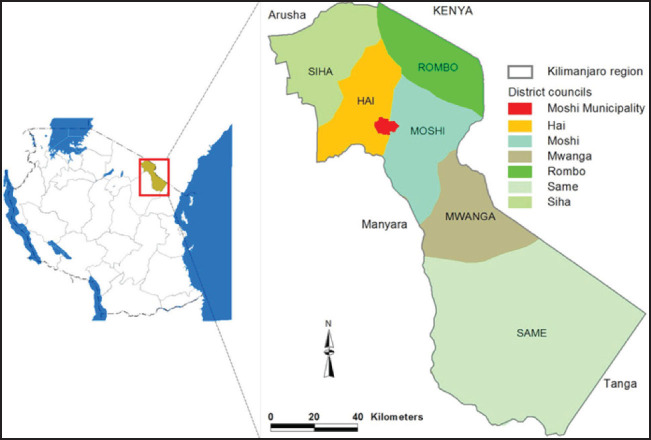
Map of the Study Area

Published reports suggest that some HCAIs can be prevented, depending on the setting, baseline infection rates, and type of infection.^13^ Health-care workers' hands are an important vehicle for transmission of micro-organism between patients and from hospital surroundings to patients.^14^ Appropriate hand hygiene practice is a key practice to prevent HCAIs. It is a cost-effective method for reduction of HCAIs and thus appropriate in resource-poor countries.^14,15^ Reports on interventions to promote hand hygiene in hospitals show that the use of alcohol based ‘hand-rub’ significantly reduces HCAIs.^13^ In 2005, the WHO launched the first Global Patient Safety Challenge with the goal of reducing HCAIs by promoting hand hygiene performance. This was followed by the establishment of a guideline on hand hygiene performance for health-care providers which aimed at improving hand hygiene practices to reduce spreading of infection to health providers and patients in 2009.^5^ The effectiveness and feasibility of criteria-based audit (CBA) in improving care in resource-limited settings has been reported from studies conducted in Jamaica and Ghana.^16^ Only a small number of studies have reported on hand hygiene performance Tanzania, and thus there is paucity of data on adherence to hand hygiene practices. In parallel with the CBA principles, success in quality of care assessment depends on accurate identification of the criteria for standard practice and appropriate case definitions. In this case, preference is given to group reflection of consensual standards rather than individual or universally defined best practices.^17–19^ The aim of this study was to assess the baseline performance and knowledge of proper hand hygiene in relation to caesarean sections and the impact on these parameters of a criterion-based audit at a tertiary health facility in Tanzania.

## METHODS

### Study Area

This study was conducted at the Kilimanjaro Christian Medical Centre (KCMC). KCMC is located on the slopes of the snowcapped Mount Kilimanjaro, located in Moshi town, the regional headquarters of Kilimanjaro region of Tanzania. KCMC is composed of a Medical University, A Research Institute and The Hospital. KCMC is a referral for over 15 million people in Northern Tanzania. The university teaching hospital is a large complex with 500-800 inpatients in 630 official beds, 90 canvas, 40 baby Incubators, 1852 students, 1300 staff and 1000 visitors and companions daily. The department of Obstetrics and Gynecology is divided into 3 units, which include Obstetric unit with 59 beds, Gynecological unit with 52 beds and labor unit with 4 delivery cubicles and 2 operation theatres. They have approximately 3,700 deliveries per year.

### Study Design and Participants

The study was a prospective, uncontrolled, before and after interventional study by criterion-based audit between 1 February and 30 June 2017. Participation was voluntary after informed consent. During the data collection period, 49 health-care workers were on duty in the labor ward. Out of the 49 health-care workers, 39 (79.6%) met the inclusion criteria and were eligible for the study. Among 39 eligible participants, 24 (61.5%), (15 doctors and 9 nurse midwives) consented and were recruited into the study. Among the 24 staff, 18 (75%) filled the knowledge test questionnaire before and after intervention, 2 (8.3%) didn't fill before intervention and 4 (16.7%) didn't fill after intervention.

### Inclusion and Exclusion Criteria

This study included doctors (Residents and Intern doctors), nurse midwives, medical attendants and intern doctors involved in caesarean sections at Kilimanjaro Christian Medical Centre (KCMC). Participants were conveniently sampled based entry criteria set. None of the departmental health workers who consented was excluded from participating in the study.

### Study Procedures

#### Criterion-based Audit

**FIGURE 2.**
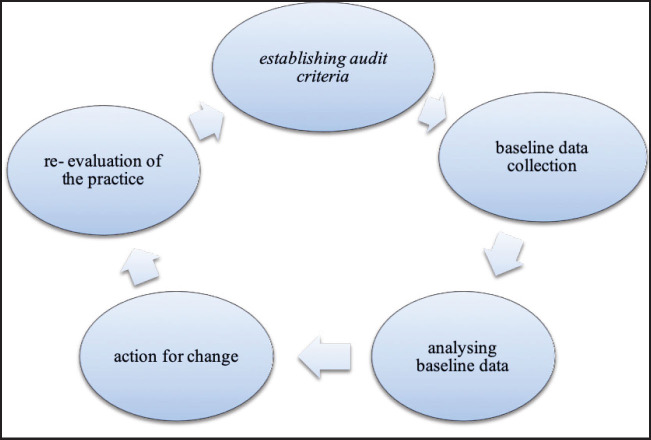
Criterion-Based Audit Cycle^21^

 A Criterion Based Audit (CBA) is a structured evaluation of practice and self-reflection to improve quality of work at health facilities. Criterion-based audit involves a review process whereby health-care workers first agree on a number of explicit and realistic criteria of good quality, adapting external guidelines to take into account the local resource context. It is important that rather than being comprehensive, the list of criteria of the CBA has to be kept short and simple to apply. Criteria are selected based on their relevance to the audited topic, the strength of the research evidence in their support, ease of measuring and the capacity of the facility in terms of human and other resources. To assess current against standard practice, an external audit assistant reviews a reasonable number of case notes for their conformity with the set criteria, and the findings are fed back to the providers. Carefully designed criterion-based audit may provide one of the most efficient methods of audit.^20^ According to Bailey and colleagues,^21^ the CBA involves 5 steps described as an audit cycle (Figure 2).

#### Step 1: Establishing Audit Criteria

Specific audit criteria of key relevance regarding hand hygiene were discussed and agreed in co-operation with 2 obstetricians and 2 nurse midwives from the labor ward at KCMC hospital. The criteria were adopted from the Tanzania National Infection Prevention and Control Standards for hospitals and WHO guidelines on hand hygiene in health care^22^ and validated Key Feature Questions for assessing knowledge of infection prevention adopted from Safe Delivery Application (SDA). Guideline contents adopted in the current study included the WHO and Tanzania national infection and prevention control in health care. These guidelines include: WHO guidelines on hand hygiene in health care: Recommended alcohol-based hands rub formulation, surgical hand preparation, hand hygiene practices among health-care workers, and adherence to recommendations, hand hygiene compliance and empowerment, WHO multimodal hand hygiene improvement strategies, Hand hygiene as quality indicator for patient safety, Hand hygiene as performance indicator, Monitoring of hand hygiene by direct and indirect methods.

Recommended methods for direct observation, Practical issues and potential barriers to optimal hand hygiene: The Tanzania national infection and prevention control in health care included: Assessment tool of infection prevention using standard and criteria applied to specific hospital areas/units, Standards and criteria applicable to specific hospital areas/units.

Adopted criteria were: Steps of Surgical hands scrub with clean running water and soap, Surgical hands preparation alcohol-based hand Surgical hand scrub time required, Short nails, no artificial nails and nail polish, 5 moments for hand hygiene, Proper wearing of sterile surgical gloves and Proper removal of sterile surgical gloves.

#### Step 2: Baseline Data Collection

Data were collected daily in a 3-week period. A minimum of 60 observations on hand hygiene were observed by using an Objective Structured Assessment of Technical Skills (OSATS) based on the first step of Criterion Based Audit cycle. Observations were performed at different duty-shifts (day, evening and night) and made on hand washing with running water and soap or by using alcohol based hand rub, wearing of gloves during and removing them after caesarean section. Knowledge on infection prevention was assessed by using structured self-administered questionnaire before and after the mid-study intervention (step 4).

#### Step 3: Analysing Baseline Data

The baseline information obtained was analysed and summarised in frequencies (counts) and proportions (percentages).

#### Step 4: Actions for Change and Implementation of Suggested Plans

The baseline results were shared with participating clinicians followed by a discussion on areas where performance on hand hygiene was observed to be sub-standard and reasons for the observed deficiencies on hand hygiene. Plans for improvement on hand hygiene were proposed by participating health-care workers. Hand hygiene and decontamination of equipment were 2 areas which were identified as in most need of improvement. A 2-day training of health-care workers was conducted for 45 minutes to 1 hour each day on strategies to improve the 2 areas of ‘weaknesses’ identified. The Safe Delivery Application (SDA) and a video on “Infection Prevention” were integrated in the training. Alcohol based hand-rub dispensers were fixed on the wall and posters on how to perform hand hygiene were posted on the wall in the theatre, labor room and triage area. Alcohol based hand-rub was distributed and filled in the dispenser for use.

#### Step 5: Re-Evaluation of the Practice

Another series of observations were performed similar to step 2 for 3 weeks after training, and findings were analysed and compared with baseline results and shared with participating mid wives and doctors.

### Data Analysis

Data were analysed using SPSS version 24 (IBM Corp., Armonk, NY, USA). Scores for knowledge test were calculated and summarised as percentages of maximum achievable score. Knowledge scores cut-off point was 50%; <50% for poor knowledge, 50%-74% for moderate knowledge and >74% for good knowledge [18]. Associations between categorical variables were analysed using the chi-square test. Fisher's exact test was used when cells had less than 5 observations. A p value of 0.05 was considered the cut-off for statistical significance.

### Ethical Considerations

Ethical review and approval to conduct this study was obtained from Kilimanjaro Christian Medical University College Research Review Committee with certificate no 2025. All participants signed informed consent after being assured anonymity and that they could withdraw from the study at any time without.

## RESULTS

Demographic characteristics of participants are presented in Table 1. The age range of the study participants was 25 to 54 years and 11 (46%) of them were between 25 and 29 years of age. Results for scores for knowledge on hand hygiene among health-care workers are summarised in Table 2. Results show an overall improvement of knowledge in the ‘poor preintervention score’ among both health worker cadres (doctors and nurse midwives) after intervention. The trend in knowledge change after intervention was similar among doctors and nurse midwives in that there was a decrease in the proportion of poor knowledge, an increase in moderate knowledge and no change for good knowledge.

**TABLE 1.** Demographic Characteristics (N=24)

Variable	n (%)
**Age (years)**	
25–29	11 (45.8)
30–34	10 (41.7)
35–54	3 (12.7)
**Sex**	
Female	10 (41.7)
Male	14 (58.3)
**Working experience**	
<12 months	11 (45.8)
≤12 months	13 (54.2)
**Profession**	
Nurse midwife	9 (37.5)
Doctor	15 (62.5)

**TABLE 2.** Knowledge Scores for Infection Prevention (n=18)

Participant Groups and Scores	Preintervention Score, n (%)	Post-Intervention Score, n (%)	Score Change (%)	*P* Value
**Doctors**				
Poor	3 (27%)	2 (18%)	−1.0 (5%)	.101
Moderate	5 (46%)	6 (56%)	+1.0 (9%)	.091
Good	3 (27%)	3 (27%)	0.0 (0%)	1.000
**Nurse midwives**				
Poor	4 (57%)	1 (14%)	−3 (43%)	.003
Moderate	3 (43%)	6 (86%)	+3.0 (43%)	.004
Good	0 (0%)	0 (%)	0.0 (0%)	1.000
**All**				
Poor	7 (39%)	3 (17%)	−4.0 (22%)	.048
Moderate	8 (44%)	12 (67%)	+4.0 (23%)	.042
Good	3 (17%)	3 (17%)	0.0 (0%)	1.000

^a^Poor ≤ 50%<Moderate ≤74%<Good

Table 3 shows results for scores on hand hygiene practices among health-care workers. Overall results show improved hygiene practice compliance as per OSATs scores at post intervention compared to baseline practices. Participants were observed with long nails and polished nails in 11(18%) and 15(25%) observations respectively at baseline. This practice at post-intervention was observed to improve by lowering to 3(5%) and 1(2%) observations of long nails and polished nails respectively (*P*=.04). Hand washing with water before procedures improved from baseline score of 44(87%) to 60(100%) post intervention (*P*=.006). Hand wash with ABHR before procedure had a low score of 7.8(13%) at baseline and this was decreased to 6(10%) post intervention (*P*=.006). However, after procedures, hand wash with ABHR showed significant improvement from the baseline score of 2((3%) to 21(35%) (*P*=.001). Also, after the procedures, hand wash with neither water nor ABHR decreased from 35 (58%) to 10 (17%)(*P*=.001). Removing gloves correctly after procedure was observed to improve form the baseline score of 20 (33%) to 29 (48%) post intervention (*P*=.01).

**TABLE 3.** Criterion-Based Audit of Hand Hygiene at Baseline and Post-Intervention Observations (N=60)

Criterion	Baseline Score	Post-Intervention Score	Score Change (%)^a^	*P Value*
**Before procedure**				
Wearing rings/bracelet/watch	6 (10%)	6 (10%)	0.0 (0%)	1.00
Long nails	15 (25%)	3 (5%)	−12.0 (20%)	.04
Polished nails	11 (18%)	1 (2%)	−10.0 (16%)	.04
Hand wash with water	43.8 (73%)	60 (100%)	+16.2 (27%)	.001
Hand wash with ABHR	7.8 (13%)	6 (10%)	−1.8 (3%)	.06
Hand wash with water or ABHR for at least 2 minutes	43.8 (73%)	44 (87%)	+0.2 (14%)	.68
Wearing sterile gloves correctly	28.8 (48%)	39 (65%)	+10.2 (17%)	.65
**After procedure**				
Removing gloves correctly	20 (33%)	37.8 (63%)	+17.8 (30%)	.01
Hand wash with water	22.8 (38%)	28.8 (48%)	+6.0 (10%)	.27
Hand wash with ABHR	2 (3%)	21 (35%)	+19.0 (32%)	.001
Hand wash with neither water nor ABHR	35 (58%)	10 (17%)	−252.0 (41%)	.001

a Negative and positive signs before scores denote decreases and increases of scores from baseline score to post-intervention score, respectively. Abbreviation: ABHR, alcohol-based hand rub

## DISCUSSION

Health-care workers’ hands are the most common vehicles for the transmission of health-care-associated pathogens between patients and within the health-care environment. Hand hygiene is the leading measure for preventing the spread of antimicrobial resistance and reducing HCAIs. However, health-care worker compliance with optimal practices remains low in most health-care settings. Good knowledge on infection prevention has an effect on infection prevention including hand hygiene. Findings from this study on health-care workers’ knowledge on infection prevention have shown that overall knowledge has improved after intervention. Our findings imply that regular attendance to infection prevention (IP) training equip health-care workers with upto date knowledge concerning prevention of infection. Similar results were reported by studies done in Ethiopia and Nigeria, where health-care workers trained on IP acquired adequate knowledge.^17–19^ Furthermore, good knowledge and practice of proper hand hygiene among health-care workers in a tertiary hospital in Western Nigeria noted to be attributed to quarterly mandatory training which was set up by the hospital infection control committee.^23^

We show low compliance of <50% at baseline hand hygiene practices during caesarean section in 8 out of the 11 criteria involved. This has adverse impact on both health-care workers and patients in relation to spread of infection. Although this study did not estimate the rate HCAIs resulting from poor hand hygiene practices, our findings is of particular concern since the impact of such low compliance would be higher rates of HCAIs transmission among patients and between patients and the health-care environment. Low hand hygiene practices is common in other countries in Africa and Asia as reported in other countries, the main reason being lack of knowledge on how proper hand hygiene is practiced, sensitisation and lack of ABHR for hand decontamination.^15,23–25^ High workload and facility ownership are additional factors linked with low compliances of hand hygiene as reported by studies in India and China, where low compliance of hand hygiene was noted more frequently in public compared to private facilities.^26–28^

Although an improvement in hand hygiene compliance was observed in the current study, little improvement was observed in washing hands with water or ABHR for at least 2 minutes and wearing sterile gloves correctly before procedure. The main reason given was absence of previous training, in congruence with results reported by other studies.^29^ Alcohol based hand rub use in health facilities is considered as the gold standard and cost effective in hand hygiene practice in the prevention of HCAIs.^5;14^ Our finding show that provision of alcohol based hand rub has contributed to the improvement of hand wash with alcohol hand rub practices especially after procedure. Similar observations were noted by Ngugi et al in Kenya, whereby health-care workers in neonatal unit had 2-fold likelihood of practising hand hygiene after than before patient care procedures.^26^ In our study, the practice of hand wash with water was more common than hand wash with ABHR during both, before and after procedures. This underscores the need for continuing training and updates of our health-care workers on infection prevention control. Alcohol-based hand rubs may be better than traditional hand washing as they require less time, act faster, are less irritating, and contribute to sustained improvement in compliance associated with decreased infection rates.^30^

Although this study might have been limited by the inherent weakness of observation bias (Hawthorn effect) where participant's behaviour change after knowing that they are being observed, yet our results provide valuable baseline information regarding hand hygiene practice among health-care workers in tertiary hospitals.

## CONCLUSION

In conclusion, we report that hand hygiene promotion, guided by health-care workers’ perceptions, supply of alcohol hand rub, training on infection prevention and how proper hand hygiene is practiced and posting of reminders on hand wash with running water and alcohol hand rub on the walls of labour ward, triage and theatre and performance feedback, is effective in sustaining compliance improvement.
